# TRAP5b and RANKL/OPG Predict Bone Pathology in Patients with Gaucher Disease

**DOI:** 10.3390/jcm10102217

**Published:** 2021-05-20

**Authors:** Margarita Ivanova, Julia Dao, Lauren Noll, Jacqueline Fikry, Ozlem Goker-Alpan

**Affiliations:** Lysosomal & Rare Disorders Research and Treatment Center, Fairfax, VA 22030, USA; jdaol@ldrtc.org (J.D.); lnoll@ldrtc.org (L.N.); jfikry@ldrtc.org (J.F.)

**Keywords:** Gaucher disease, osteoporosis, TRAP5b, OPG, RANKL, biomarker, lyso-Gb1, bone

## Abstract

Background and objective: Bone involvement occurs in 75% of patients with Gaucher disease (GD), and comprises structural changes, debilitating pain, and bone density abnormalities. Osteoporosis is a silent manifestation of GD until a pathologic fracture occurs. Thus, early diagnosis is crucial for identifying high-risk patients in order to prevent irreversible complications. Methods: Thirty-three patients with GD were assessed prospectively to identify predictive markers associated with bone density abnormalities, osteopenia (OSN), and osteoporosis (OSR). Subjects were categorized into three cohorts based on T- or Z-scores of bone mineral density (BMD). The first GD cohort consisted of those with no bone complications (Z-score ≥ −0.9; T-scores ≥ −1), the second was the OSN group (−1.8 ≥ Z-score ≥ −1; −2.5 ≥ T-score ≥ −1), and the third was the OSR group (Z-score ≤ −1.9; T-scores ≤ −2.5). Serum levels of TRAP5b, RANKL, OPG, and RANK were quantified by enzyme-linked immunosorbent assays. Results: TRAP5b levels were increased in GD patients, and showed a positive correlation with GD biomarkers, including plasma glucosylsphingosine (lyso-Gb1) and macrophage activation markers CCL18 and chitotriosidase. The highest level of TRAP5b was measured in patients with osteoporosis. The elevation of RANKL and RANKL/OPG ratio correlated with osteopenia in GD. Conclusion: TRAP5b, RANKL, and RANKL/OPG elevation indicate osteoclast activation in GD. TRAP5b is a potential bone biomarker for GD with the ability to predict the progression of bone density abnormalities.

## 1. Introduction

Gaucher disease (GD), the most common lysosomal storage disorder, is caused by a deficiency of the enzyme glucocerebrosidase (GCase) and the progressive accumulation of its substrate, glycosylceramide (GC), in various tissues and organs of the reticuloendothelial system [1]. GD affects monocyte lineage cells, primarily the macrophages, which play an essential role in bone metabolism, osteoclast differentiation, osteoclasts-osteoblasts interactions, and bone remodeling. Bone involvement in GD ranges from osteonecrosis to reduced bone density and developmental and structural bone abnormalities.

The progressive bone disease occurs in 75% of patients with type 1 GD, and signs and symptoms include structural bone changes, debilitating bone pain, and osteoporosis [2]. In addition to the extensive inflammatory response against GC and its toxic metabolite glucosylsphingosine (lyso-Gb1), GD bone pathology is possibly the result of the alterations in osteoclast function and osteoblast participation in bone remodeling and osteoclast differentiation [3]. Moreover, local GC accumulation affects osteoclast-osteoblast communication and mediates bone abnormalities [4,5,6].

Reduced bone density leads to progressive osteopenia and osteoporosis, and the aberrations in bone structure lead to abnormal vertebral remodeling and bone modeling, including Erlenmeyer flask deformity. Other bone structural abnormalities in GD include osteonecrosis and lytic lesions [3,7].

Tartrate-resistant acid phosphatase (TRAP) is an enzyme coded by *ACP5*, and is expressed in osteoclasts, macrophages, and dendritic cells. Two isoforms of TRAP circulate in the blood, TRAP5a secreted from macrophages and dendritic cells, and TRAP5b from osteoclasts. TRAP5b is a marker of osteoclast activity and indicators of bone resorption [8]. Moreover, the activator of TRAP5b expression, cathepsin K, is highly expressed in osteoclasts and has been shown to participate in bone resorption in GD [9]. Although elevated TRAP5b has previously been reported in GD, the limited number of patients included in this study prevented any further conclusions about its significance from being reached [10].

Among the essential factors that regulate bone turnover are the receptor activator of NF-kB (RANK), its ligand (RANKL), and osteoprotegerin (OPG), the receptor that binds to RANKL [9,11,12]. The cellular response to RANKL is contingent on the level of its receptor RANK and the presence of OPG [11]. RANKL and OPG are primarily involved in maintaining bone density. Given that bone is an organ with a slow turnover, and that bone mineral density (BMD) measurement in the short term does not provide enough information about prognosis, biomarkers will be valuable for the early assessment of bone density abnormalities.

## 2. Materials and Methods

### 2.1. Subjects

The study was conducted under an IRB approved protocol (NCT04055831) and included 33 subjects with GD (eight males and 25 females, age range 18 to 68 years, mean 41 ± 15) and 15 healthy controls (nine males and six females with an average age of 48 ± 11 years) (Supplemental Table S1). Ethics committees and data protection agencies approved the clinical protocol, and all subjects provided written informed consent for the collection of samples and analysis of their data. GD diagnosis was based on GCase residual activity and *GBA* molecular analysis. Participants were further categorized into three cohorts based on the T- or Z-score of BMD. The cohort designated ‘normal’ (N) included nine subjects without any bone complications and a normal BMD with a Z-score ≥ −0.9 or T-score ≥ −1. The osteopenia (OSN) group included 10 subjects with a Z score of −1 to −1.8 or a T-score of −1 to −2.5. The osteoporosis (OSR) group included 14 subjects with a Z score ≤ −1.9 or a T-score ≤ −2.5 (Table 1 and Supplemental Table S1).

### 2.2. The Clinical Features of Bone Disease in GD Cohorts

Details of medical history with bone disease characteristics such as bone surgery, pathologic fractures, bone pain, bone marrow infiltration, EM-flask deformity, and osteonecrosis are listed in Table 1 and Supplemental Table S1. Demographic characteristics, genotypes, and relevant molecular analyses are summarized in Supplemental Tables S2 and S3.

### 2.3. Measurement of Biomarkers in Plasma Samples

Blood samples were collected in EDTA tubes. Plasma levels of bone markers were measured using commercially available ELISA kits. The concentration of TRAP5b was measured in 50 µL of plasma using TRAP5b ELISA kit (Quidel, San Diego, CA, USA). Recombinant TRAP5b was used for calibration, and the range of the assay was 0–16.5 U/L. The concentration of OPG and RANKL was measured in 100 µL of plasma using OPG and RANKL ELISA kits (Origene Technologies Inc., Rockville, MD, USA). Recombinant OPG was used as a standard, and the range of the assay was 0–6000 pg/mL. The RANKL range of the assay was 0–5000 pg/mL. The RANK concentration was measured in 100 µL of plasma using the RANK ELISA kit (Thermo Fisher Scientific, Waltham, MA, USA). Recombinant RANK was used as a standard, the range of the assay was 0–10 ng/mL, and the analytical sensitivity was 0.054 ng/mL.

### 2.4. Statistical Analysis

Statistical analysis was performed using Graph Prizm (GraphPad, San Diego, CA, USA). Differences between the two groups were tested by Student’s *t*-test or F-test. The groups were compared using one-way analysis of variance (ANOVA), followed by Brown-Forsythe, Bartlett’s multiple-comparison, and Kruskal-Wallis tests. Pearson’s test, one or two tails, was used for correlation analysis. The value of *p* < 0.05 indicated a statistically significant result.

## 3. Results

### 3.1. Bone Pain Is Associated with Osteopenia and Osteoporosis in GD

Overall and individual characteristics of bone involvement in subjects with GD are given in Table 1 and Supplemental Table S1. Bone pain was more common in the OSR group (71%) compared to the N (44%) and OSN groups (40%) (Table 1). Increased frequency of pathological bone fractures and bone surgeries correlated with the progression of OSN and OSR (Table 1). However, the incidence of bone marrow infiltration, EM-flask deformity, and osteonecrosis were not associated with abnormal bone density, or with OSN or OSR pathology (Table 1).

### 3.2. TRAP5b Is Increased in GD and Correlates with Osteoporosis

**Figure 1:**
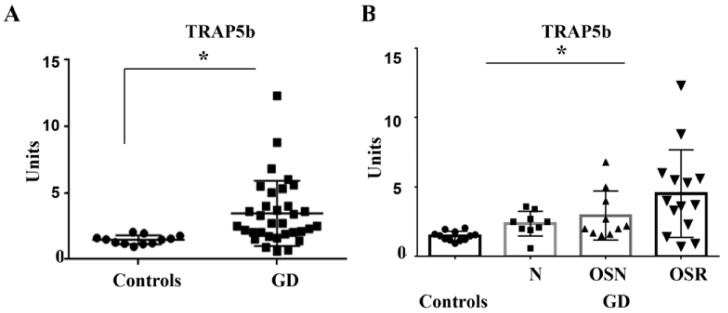
Plasma TRAP5b level. (**A**) TRAP5b level, GD (Gaucher disease) mean 3.4 ± 0.4, *n* = 33 vs. control mean 1.4 ± 0.1, *n* = 10, *p* < 0.05 unpaired *t*-test and F test. (**B**) TRAP5b concentrations in control and GD cohorts. GD and no bone complication (N, mean 2.6 ± 0.63), osteopenia (OSN, mean 3.1 ± 1.8), and osteoporosis (OSR, mean 4.2 ± 2.2). * *p* < 0.05; ANOVA, Brown-Forsythe, and Bartlett’s multiple-comparison test. Data are means ± SEM. Measurements are in units/50 μL.

TRAP5b is a marker of the number of active osteoclasts and an indicator of bone resorption. To examine the role of TRAP5b in GD, the peripheral levels of TRAP5b were measured. TRAP5b was significantly higher in the GD group than in the healthy controls (Figure 1A). Further analysis demonstrated that TRAP5b correlated with the progression of osteopenia towards osteoporosis (Figure 1B). TRAP5b was significantly higher in the OSR than in the OSN and N cohorts.

Furthermore, the finding that TRAP5b is elevated in GD patients remained unchanged after stratifying by gender (Supplemental Figure S1A). eight out of ten GD women and three out of four GD men with osteoporosis showed elevated levels of TRAP5b. (Supplemental Figure S1A). Taken together, these data indicate that plasma TRAP5b may be a clinically relevant marker for the evaluation of bone resorption in GD patients. There was no correlation between TRAP5b levels for different treatment modalities, enzyme replacement therapy (ERT), substrate reduction therapy (SRT), or treatment duration.

### 3.3. RANKL, Not RANK Is Elevated in GD and Correlates with Osteopenia

**Figure 2:**
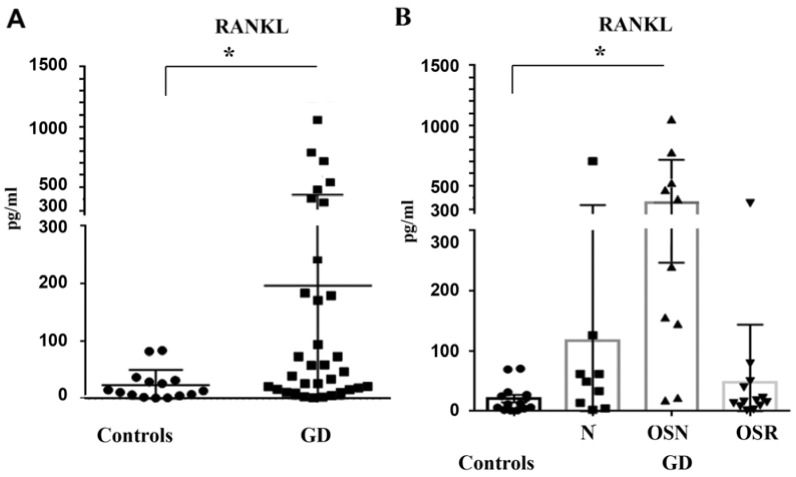
Plasma RANKL level. (**A**) RANKL levels, control vs. GD. Statistical analysis using unpaired *t*-test and F test to compare variance demonstrated a significant difference between control (mean 20 *±* 5.8, *n* = 15) and GD cohorts (mean 166 ± 67.9, *n* = 33). * *p* < 0.05. (**B**) RANKL concentrations in control subjects and GD with no bone complication (N, mean 117 ± 74), osteopenia (OSN, mean 362 ± 118), and osteoporosis (OSR, mean 49 ± 26). * *p* < 0.05 ANOVA test, Tukey’s multiple comparisons test control vs. OSN and control vs. OSR.

RANKL is highly expressed by osteoblasts and osteocytes. RANKL binds to its receptor (RANK) and activates osteoclasts’ differentiation and maturation, favoring bone resorption [11,13]. Analysis of control plasma and plasma from GD subjects demonstrated that the level of RANKL was significantly higher in GD (Figure 2A). Comparing RANKL in controls and GD cohorts showed a significant increase in RANKL level in the OSN cohort compared with the N and OSR cohorts (Figure 2B). RANKL level was elevated in GD females with OSN (5/8) and in two women (2/10) with OSR (Supplemental Figure S1B). Three males with GD presented with the highest level of RANKL, one patient without bone complications and two patients with OSN. Five subjects with osteoporosis were treated with denosumab, a human monoclonal antibody against the RANK ligand. RANKL level was elevated in one of five patients treated with denosumab (Supplemental Figure S1B). Correlation between type or duration of Gaucher disease specific treatment and RANKL was not observed. Overall, these results show that plasma RANKL is a potential marker for OSN in GD.

**Figure 3:**
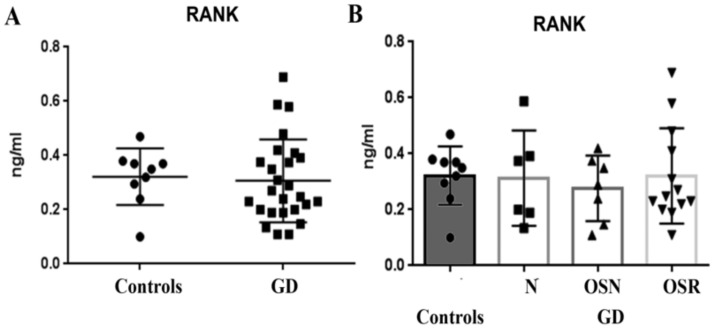
Circulating RANK levels in patients with GD. (**A**) RANK levels, control vs. GD group (mean 0.30 ± 0.02, *n* = 30). (**B**) RANK concentrations in control (mean 0.32 ± 0.03, *n* = 10) and GD groups N, OSN, and OSR. Data are means ± SEM. Measurements are in ng/mL.

RANKL binds to the receptor activator of NF-kB (RANK) to induce osteoclastogenesis [11]. RANK is mainly expressed in osteoclast precursors, mature osteoclasts, dendritic cells, macrophages, and microglia. Surprisingly, despite being a membrane receptor, RANK was detectable in the serum of GD subjects. However, the measurement of plasma RANK showed no differences between the controls and GD patients (Figure 3A). Further analysis in three different GD cohorts did not show an association between RANK and the progression of bone density abnormalities (Figure 3B).

### 3.4. Elevated OPG Does Not Correlate with OSN or OSR in GD

**Figure 4:**
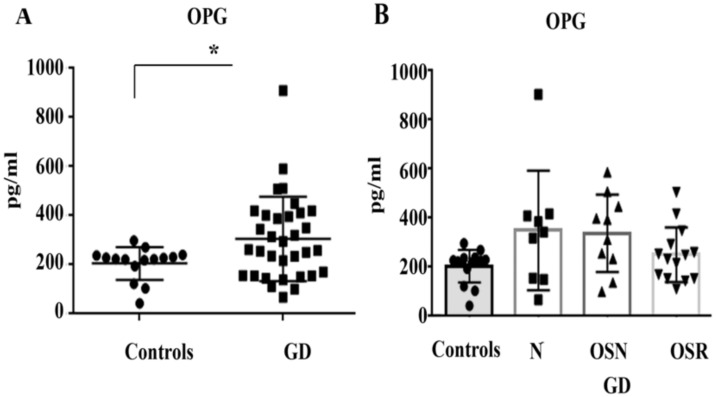
Plasma OPG (osteoprotegerin) concentrations. (**A**) OPG level, control (mean 206 ± 17, *n* = 10) vs. GD (mean 307 ± 30, *n* = 33). * *p* < 0.05 unpaired *t* test and F test. (**B**) OPG concentrations in control subjects and GD with no bone complication (N), osteopenia (OSN), and osteoporosis (OSR).

The decoy receptor for RANKL (OPG) is produced by osteoblasts. OPG binds RANKL and blocks RANKL activation, reducing the number of osteoclasts [14]. Plasma OPG was elevated in 15 out of 33 (46%) GD patients, and the average of OPG in GD was higher compared with healthy controls (Figure 4A). Further analysis, however, demonstrated that OPG did not correlate with osteopenia or osteoporosis (Figure 4B). The majority of patients with the highest level of OPG belonged to two cohorts: no bone complication (5 out of 8 patients) or OSN (5 out of 9 patients). After stratification for gender and treatment, there was no difference in OPG levels. (Supplemental Figure S1C).

### 3.5. RANKL/OPG Ratio Is Higher in Patients with GD

**Figure 5:**
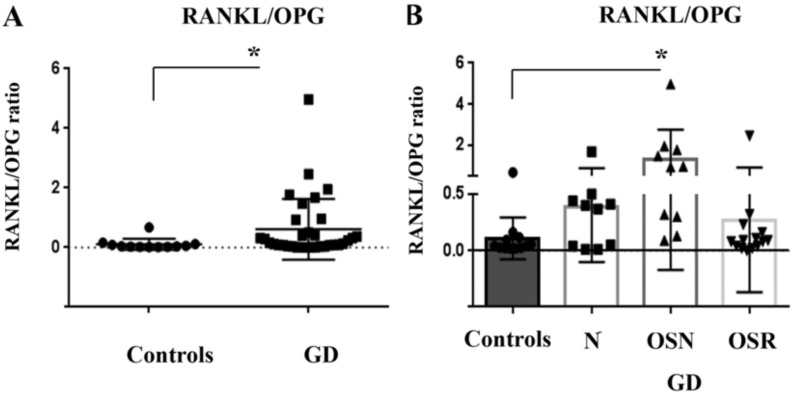
RANKL/OPG ratio. (**A**) Comparing RANKL/OPG ratio in control (mean 0.10 ± 0.06, *n* = 10) vs. GD cohorts (mean 0.60 ± 0.17, *n* = 33). F test significant *p* < 0.05 (**B**) RANKL/OPG ratio in control group (mean 0.11 ± 0.08, *n* = 10); GD-N (mean 0.39 ± 0.17, *n* = 9); GD-OSN (mean 1.3 ± 0.46, *n* = 10); and GD-OSR (mean 0.27 ± 0.170, *n* = 14) groups. Data are means ± SEM. ANOVA test, Bartlett’s multiple-comparisons, and Kruskal–Wallis tests (* *p* < 0.05) showed significant differences between groups. Tukey’s multiple-comparison test demonstrated significant differences between control vs. OSN groups.

Alterations of the RANKL/OPG balance have been characterized in a wide range of bone diseases, including osteoporosis [13]. Therefore, in addition to RANKL and OPG analysis, we calculated the RANKL/OPG ratio. Similar to RANKL and OPG, the RANKL/OPG ratio was higher in GD compared to controls (Figure 5A). Further analysis showed an increased RANKL/OPG ratio in the OSN cohort. Tukey’s multiple-comparison test demonstrated significant differences between control vs. OSN groups (Figure 5B). The observation that RANKL/OPG ratio is higher in GD patients, especially in patients with osteopenia, remained the same for males and females (Supplemental Figure S1D).

### 3.6. The Relationship between TRAP5b, RANKL, and OPG in GD

**Figure 6:**
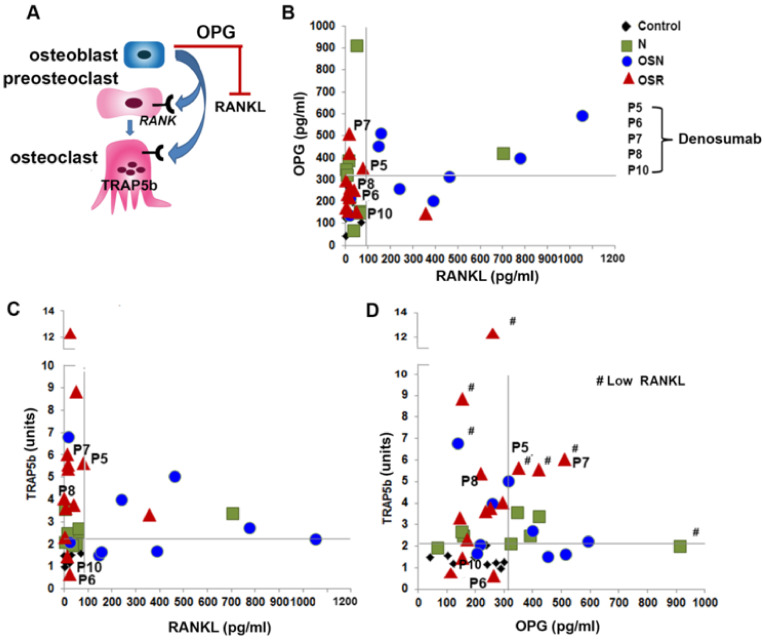
TRAP5b, RANKL, and OPG correlation in GD. (**A**) The RANKL-OPG-RANK pathway activates bone resorption and osteoclast differentiation. Osteoblasts express RANKL and OPG. Preosteoclasts express RANK, which is activated by RANKL. OPG neutralizes RANKL. TRAP5b, expressed by osteoclasts, is a marker of osteoclast activity. (**B**–**D**) Scatterplot analysis of correlation of OPG and RANKL (**B**), TRAP5n and RANKL (**C**), and TRAP5b and OPG (**D**). P5, P6, P7, P8, and P10 represent patients treated with denosumab. Healthy control (black diamond). GD cohorts: N (green), OSN (blue), and OSR (red). The graphs are divided into quadrants and demonstrate: left bottom (range of healthy controls), left top (higher than healthy control only in the vertical axis), right bottom (higher than healthy control only in the horizontal axis), and right top (higher than healthy control in both the vertical and the horizontal axis).

Correlation analysis of RANKL/OPG/TRAP5b levels in GD patients with no bone complications (N) showed an increased level of TRAP5b and OPG, but not RANKL. From the GD-N cohort, only one patient had an elevated level of RANKL; however, this patient had a high level of OPG and a TRAP5b level comparable to the average control level (Figure 6A,B and Supplemental Figure S2).

Scatterplot analysis of the relationship between RANKL and OPG demonstrated that some samples from the OSN cohort with higher RANKL also showed increased OPG levels (Figure 6A and Supplemental Figure S2). Since RANKL promotes osteoclastogenesis, the elevated level of serum RANKL and the decreased level of OPG indicate accelerating osteoclast activation, resulting in osteoporosis progression. Scatterplot TRAP5b vs. RANKL demonstrates that TRAP5b is higher in the OSN cohort with an elevated RANKL and normal OPG (Figure 6B,C and Supplemental Figure S2). Thus, these data suggest that an increased level of OPG inhibits osteoclast activity in GD patients with osteopenia.

In the GD cohort with OSR, the RANKL and OPG levels were similar to control and GD-N cohorts, except for one patient with a high RANKL and three patients with high OPG only (Figure 6B and Supplemental Figure S2). There was no direct correlation between TRAP5b and RANKL or OPG levels in GD-OSR (Figure 6C,D). Because osteoclast activity is significantly greater than osteoblast activity in osteoporotic tissue [15], the significantly increased level of TRAP5b, but not RANKL or OPG, might be associated with a shift to osteoporosis in GD.

Among the five patients treated with denosumab, two (P6 and P10) had normal RANKL, OPG, and TRAP5b levels (Figure 6B–D). Three (P5, P7, P8), meanwhile, had an elevated TRAP5b, and two patients had increased OPG.

### 3.7. TRAP5b Positively Correlates with GD Biomarkers: CCL18, Chitotriosidase, and Lyso-Gb1

**Figure 7:**
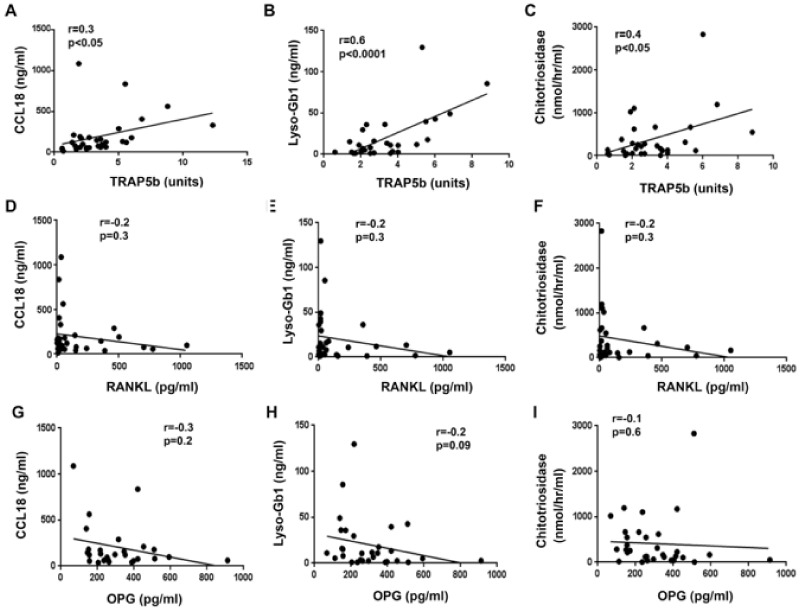
TRAP5b, RANKL, OPG correlation with biomarkers: CCL18, lyso-Gb1, and chitotriosidase in all GD patients. Scatterplot analysis of correlation of TRAP5b and CCL18 (**A**), TRAP5b and lyso-Gb1 (**B**), TRAP5b and chitotriosidase (**C**), RANKL and CCL18 (**D**), RANKL and lyso-Gb1 (**E**), RANKL and chitotriosidase (**F**), OPG and CCL18 (**G**), OPG and lyso-Gb1 (**H**), and OPG and chitotriosidase (**I**). *p*-values: statistical comparison was determined via Pearson’s two tail linear regression correlation analysis.

We next analyzed the correlation between circulating TRAP5b, RANKL, and OPG, and GD biomarkers chitotriosidase (CHITO), lyso-Gb1, and chemokine ligand 18 (CCL18) in all GD patients. CCL18 and CHITO are secreted by activated macrophages, and lyso-Gb1 represents the circulating metabolite derived from the deacylation of Gb1 [16,17,18]. A positive linear correlation was observed between CHITO, lyso-Gb1, and CCL18 (Supplemental Figure S3). TRAP5b showed a significant positive correlation with CCL18, lyso-Gb1, and CHITO (Figure 7 and Supplemental Figure S4). Furthermore, a negative correlation was observed between OPG and CHITO in the majority of GD patients with abnormal bone density (OSN and OSR) (Supplemental Figure S4), with the exception of two OSR patients who had incredibly high levels of CHITO and an elevated level of OPG (Supplemental Figure S4I). A negative correlation was observed between OPG and lyso-Gb1 in GD patients with OSN (Table 2). In comparison, there was no correlation between RANKL and GD biomarkers.

## 4. Discussion

Progressive bone disease is one of the primary untreated aspects of Gaucher disease. The majority of GD patients had structural bone involvement, and 43% had bone pain [19]. Bone metabolism, including turnover, remodeling, and mineralization, are affected in GD. One of GD’s early signs involving bone pathology is the ‘Erlenmeyer flask’ deformity that affects long bones and abnormality of bone modeling. Later, the majority of GD patients develop skeletal complications, including osteopenia and osteoporosis [3]. GD patients with skeletal involvement could be asymptomatic or present symptoms such as pain, pathological fractures, cystic changes, or osteonecrosis. ‘Erlenmeyer flask’ deformity occurs during tubular and long bone growth [20], and can be the initial diagnostic sign in many patients. With GD, in our cohort, the ‘Erlenmeyer flask’ deformity did not correlate with OSN or OSR, suggesting different pathological pathways leading to abnormal bone remodeling and bone mineralization. However, in our study, an increasing number of GD patients with bone pain and bone fractures correlated with osteoporosis, suggesting similar underlying mechanisms for both. As is well known, multiple chronic immune and inflammatory disorders are associated with bone density abnormalities and accompanying pain [21].

While bone involvement is common in GD, there are no peripheral bone-related biomarkers in clinical use that could assist with therapeutic planning and clinical management. Our study’s main conclusion is that TRAP5b is a biomarker that correlates with the progression of osteopenia to osteoporosis in GD. Excess osteoclastic bone resorption over osteoblastic bone formation leads to bone mineral loss and the development of osteopenia and osteoporosis [20]. The bone marker, TRAP5b, is a marker of osteoclast activation and reflects the number of active osteoclasts [8,22]. Two isoforms of TRAP circulate in the blood, TRAP5a and TRAP5b. TRAP5a is a biomarker of systemic inflammation [23], and TRAP5b is a biomarker of bone resorption. The development of specific TRAP5a and TRAP5b antibodies has made it possible to separate clinically relevant biomarkers for osteoclasts and inflammatory macrophages [24].

Interestingly, total TRAP, not TRAP5b, has been used as a biomarker for GD along with angiotensin-converting enzyme (ACE), CHITO, and ferritin, all of which are markers for activated macrophages [25]. Moreover, total TRAP, along with CHITO, ferritin, and ACE, is included in routine clinical monitoring of GD activity [26]. Our results demonstrate that higher TRAP5b levels correlate with OSN (3.1 ± 1.8) and OSR (4.2 ± 2.2) progression in GD. Similar to TRAP5b, cathepsin K is also secreted predominantly by activated osteoclasts [27,28]. Moreover, two studies demonstrated that cathepsin K increases in GD1 [29,30]. Thus, enhanced TRAP5b expression and cathepsin K activation confirm osteoclast activation in GD patients. Overall, our findings suggest that serum TRAP5b is a promising new biomarker with clinical relevance in GD, and evaluation of osteopenia–osteoporosis progression.

Circulating TRAP5b positively correlates with clinical biomarkers of GD pathology: CCL18, lyso-Gb1, and CHITO. We think that this finding is relevant to understanding the pattern of events that occurs in GD bones. Because the significant correlation was verified between TRAP5b and lyso-Gb1, and correlations between lyso-Gb1, CHITO are CCL18 have been observed in several clinical studies [18], we postulate that lyso-Gb1 is the primary contributor to TRAP5b activation, not CHITO or CCL18. A possible mechanism underlying activation expression of TRAP5 in osteoclasts is Gb1 accumulation in bone marrow cells, including monocytes/macrophages precursors cells, and osteoclasts. An analysis of osteoblasts differentiated from a GD1 patient’s bone marrow demonstrated that exogenous lyso-Gb1 reduced mesenchymal cell viability for osteoblast differentiation and reduced osteoblast calcium deposition [6]. At the same time, an analysis of osteoclasts showed an increase in osteoclast generation of 37% in GD. Thus, increased osteoclast number and activity with reducing osteoblast activity lead to osteoclast/osteoblast disbalance in GD [6]. The alternative pathway of osteoclast activation is via inflammatory pathways that lead to an augmented and systemic loss of bone mineral density [31]. A wide range of in vitro studies have shown that lyso-Gb1 is a pro-inflammatory agent in immune cells, including chronic B-cell and T-cell activation and gammopathy [32,33,34]. We suggest that TRAP5b could be used along with other GD biomarkers to assess bone density abnormalities and response to therapy.

The level of RANKL and OPG are essential factors that determine the number and activity of osteoclasts [11,35,36]. RANKL binds to RANK on the surface of the osteoclast precursor or mononuclear osteoclast and promotes osteoclast differentiation and maturation. OPG binds to RANKL and inhibits osteoclast differentiation. RANK is expressed in osteoclast precursors, mature osteoclasts, dendritic cells, macrophages, and microglia. The RANKL is highly expressed by osteoblasts, and to a much lesser degree in osteocytes. OPG is mainly expressed by osteoblasts [35]. Mature monocytes and macrophages have the ability to differentiate into osteoclasts, but these cells also secrete factors that impact osteoblast activity [37,38,39]. Because cells of monocytes/macrophage lineage are primarily affected in GD, it is not unexpected that bone morphogenesis and remodeling are impaired [3,40]. GD patients with active bone disease formed more osteoclasts than GD patients without bone disease [6,40,41]. Moreover, an in vitro inhibitory GD model that demonstrated that PBMC differentiated into osteoclasts at a higher rate support the finding that GD patients with active bone disease formed more osteoclasts [41]. Thus, the altered RANKL/RANK/OPG triad plays an integral role in GD bone pathology [3,18].

In the present study, RANKL was elevated in 51% of the patients with GD. However, elevated plasma RANKL only correlates with osteopenia, and not osteoporosis, suggesting that the acceleration of osteoclast differentiation occurs before the onset of osteoporosis. This finding may also represent the activation of osteoclastic bone resorption in patients with osteopenia. Furthermore, the majority of GD patients had an average serum OPG level, and only 41% presented with an elevated OPG. Patients with a high level of OPG either belonged to the N or OSN cohorts. This finding suggests that in the early stages of bone disease, OPG is expressed and inhibits RANKL activity. Overall, our data fit with a previous study that demonstrated the normal values for OPG in GD type 1 [42]. Accumulated findings indicate that OPG-related osteoclast activity is not a major mechanism of bone pathology in GD patients with relatively mild form [42]. However, there is still controversy regarding RANKL/OPG and its effects on GD bone pathology [43]. The genetic variability of OPG and RANK genes in GD may also play a role in the GD bone pathology with its response to treatment. Similar to the RANKL data, RANKL/OPG level was mostly elevated in the OSN group and positively correlated with RANKL. The higher level of RANKL and RANKL/OPG in patients with osteopenia has been observed in postmenopausal women [44]. RANKL/OPG ratio is often used as a biomarker of bone pathology, which reflects a balance of bone formation and resorption.

The close relationship between osteoporosis and chronic inflammation has been studied for many years. The most detailed observations of osteoclast-mediated bone loss during chronic inflammation are described for autoimmune rheumatic diseases. Osteoclast-mediated resorption at the interface between synovium and bone is responsible for the joint erosion seen in patients suffering from inflammatory rheumatoid arthritis (RA) [45]. Inflammatory cytokines (tumor necrosis factor-alpha (TNF*α*) and interleukins (IL-1, and Il-6)) may drive osteoclastic bone loss [45,46]. Synovial macrophages in RA overexpress these inflammatory cytokines, including TNF*α*, which has particularly pervasive effects on osteoclastogenesis by promoting RANKL production [46,47]. RANKL, in turn, promotes differentiation of synovial macrophages into osteoclasts [48]. Because Gb1 and lyso-Gb1 induce expression of pro-inflammatory cytokines in macrophages, including TNF*α*, Il-6, and monocyte chemoattractant protein 1 (MCP-1) [18], we suggest that inflammatory cytokines contribute to RANKL/OPG imbalance and thus promote osteoclastic bone resorption. The bone pain that also correlates with bone density abnormalities suggests the contribution of a systemic inflammatory response to GD-related bone disease [49].

A relatively small number of subjects and small cohort numbers for each GD is one of the limitations of this study, and thus, the results of linear regression analysis should be interpreted with caution. Also, in this study, we did not take into account the age of disease onset, disease course, or duration of GD therapy, which could have contributed to current observations in OSN and OSR cohorts.

ERT leads to a substantial improvement of hematological manifestation in GD; however, bone involvement is refractory to therapy [50]. Limited studies have evaluated bone biomarkers in untreated vs. ERT treated patients with inconsistent results. The lack of differences in serum OPG levels between naïve and ERT-treated patients suggested that OPG-related osteoclast activity may not be a significant contributor to GD bone pathology [42]. However, in another study, there were decreased OPG and RANKL/OPG levels in GD [10]. Our group has previously demonstrated that plasma RANKL and OPG levels decreased in SRT-treated patients over time, but RANKL/OPG did not change with the treatment status [51].

There is no standard treatment for osteopenia–osteoporosis progression in GD. The most common medications for osteoporosis and others bone diseases, nitrogen-containing bisphosphonates (N-BPs), prevent bone mass loss by inhibiting osteoclast resorption via inhibition of farnesyl pyrophosphate synthase [52]. N-BPs, calcium, and vitamin D with a healthy lifestyle are standard recommendations for GD patients, especially for young patients [53,54]. Unfortunately, no clinical study using N-BPs in GD has been completed. While there are individual case reports, limited small patient series, and consensus statements on the use of N-BPs, there has not yet been a systemic study of this treatment modality in GD [53,55,56]. Emerging osteoporosis therapies utilize novel mechanisms, including monoclonal antibodies against RANKL, DKK1, and sclerostin [57]. The antibody against RANKL, denosumab, prevents osteoclast development through RANKL inhibition. In this study, five patients with OSR have been treated with denosumab; among those, four had normal RANKL, and one had borderline levels after receiving denosumab. Nevertheless, the majority of GD patients with elevated RANKL levels were patients with OSN. Currently, whether patients with osteopenia and an elevated RANKL but normal OPG levels have a higher risk of developing osteoporosis is not known. Similarly, the effects of denosumab in GD-related osteoporosis is yet to be studied [20].

This study furthers clinical knowledge regarding the circulating biomarkers (TRAP5, RANKL, RANK, and OPG), bone density, and osteoporosis progression in GD. We provide the first evidence that TRAP5b is a potential bone biomarker for GD with the ability to predict OSN and OSR progression. The pattern of elevation of RANKL and OPG provides additional evidence for the role of osteoclastic bone resorption in GD.

## Figures and Tables

**Table 1:** The clinical features of bone disease in GD (Gaucher disease) cohorts: no bone complications (N), osteopenia (OSN), and osteoporosis (OSR).

	N	OSN	OSR
T-score (average ± STDEV)	0.03 ± 0.2	−1.07 ± 0.2	−2.96 ± 0.8
Z-score (average ± STDEV)	−0.2	−1.6	−2.73 ± 0.4
Bone pain	4/9 (44%)	4/10 (40%)	10/14 (71%)
Bone surgery	0/9 (0%)	1/10 (10%)	6/14 (42%)
Pathologic fractures	0/9 (0%)	2/10 (20%)	3/14 (21%)
Bone marrow infiltration	7/9 (77%)	7/10 (67%)	8/14 (57%)
EM-flask deformity	5/9 (55%)	3/10 (30%)	8/14 (57%)
Cystic changes	0/9 (0%)	0/10 (0%)	1/14 (7%)
Osteonecrosis	3/9 (33%)	1/10 (10%)	4/14 (28%)

**Table 2:** TRAP5b, RANKL, OPG correlation with CCL18, lyso-Gb1, and chitotriosidase (CHITO) in N, OSN, and OSR cohorts. Statistical comparisons were determined via Pearson’s one tail linear correlation analysis. * Statistical analysis included GD patients with abnormal bone density (OSN and OSR), two OSR outliers were removed.

		TRAP5b	OPG	RANKL
CCL18	N	N	N	N
	OSN	positive correlation (R = 0.7; *p* = 0.02)	N	N
	OSR	positive correlation (R = 0.5, *p* = 0.03)	N	N
CHITO	N	N	N	N
	OSN	positive correlation (R = 0.8, *p* = 0.006)	negative correlation (R = −0.6, *p* = 0.03)	N
	OSR	positive correlation (R = 0.2, *p* = 0.05)	OSN + OSR (R = −0.4, *p* = 0.02 *)	N
Lyso-Gb1	N	N	N	N
	OSN	positive correlation (R = 0.7, *p* = 0.02)	negative correlation (R = −0.6, *p* = 0.03)	N
	OSR	positive correlation (R = 0.6, *p* = 0.02)	N	N

## Data Availability

Not applicable.

## Supplementary Materials

The following are available online at https://www.mdpi.com/article/10.3390/jcm10102217/s1, Figure S1: TRAP5b and RANKL concentrations in controls and GD females vs. males, Figure S2: Scatterplots represent the correlation between RANKL, OPG, and TRAP5b, Figure S3: Scatterplots represent the correlation between Lyso-Gb1, CCL18, and chitotriosidase (CHITO) in all GD patients. Figure S4: Scatterplots represent the correlation between Lyso-Gb1, CCL18, chitotriosidase (CHITO) and bone biomarkers, Table S1: GD with bone pathology characteristics, Table S2: Demographic characteristics, Table S3: Genotype summary.
