# Supplementary material for: TRAP5b and RANKL/OPG Predict Bone Pathology in Patients with Gaucher Disease
Source: J Clin Med. 2021 May 20;10(10):2217. doi: 10.3390/jcm10102217 (PMC8160801; doi:10.3390/jcm10102217)
Supplement: Supplementary file 1 [file jcm-10-02217-s001.zip › jcm-1204902-supplementary.pdf]

Journal of
Clinical Medicine

TRAP5b and RANKL/OPG predict bone pathology in patients with Gaucher disease.

Supplementary Materials:

Table S1. GD with bone pathology characteristics. (* Minimal dark marrow; # Patchy dark marrow).

	**BMD Score**		**Splenectomy**	**Bone surgery**	**Bone pain**	**Bone marrow infiltration**	**EM-flask deformity**	**Cystic Changes**	**Pathologic fractures**	**Osteonecrosis**	**Treatment and Duration**	
	**Z-**	**T-**									ERT/SRT	years
						No bone complication						
**1**	**-0.2**		**No**	**No**	**Yes**	**Yes**	Yes	No	No	No	ERT	5-10
2		-0.2	No	No	Yes	Yes	Yes	No	No	Yes	ERT	<10
3	-0.9		No	No	No	Yes #	Yes	No	No	Yes	SRT	<10
4	-0.9		No	No	No	Yes #	Yes	No	No	No	ERT	5-10
5	-0.8		No	No	No	No	No	No	No	No	SRT	<10
6	0.0		No	No	No	No	No	No	No	No	ERT	<10
7	0.0		No	No	Yes	Yes	No	No	No	Yes	ERT	<10
8	0.2		No	No	No	Yes	No	No	No	No	SRT	5-10
9	0.6		No	No	Yes	Yes	Yes	No	No	No	ERT	1-5
						Osteopenia						
1	-1.3		No	Yes	No	Yes	Yes	No	No	No	ERT	1-5
2		-1.6	No	No	Yes	Yes	Yes	No	Yes	Yes	ERT	<10
3	-1.5		No	No	No	No	No	No	No	No	ERT	5-10
4	-1.1		No	No	No	Yes	No	No	No	No	SRT	<10
5	-1		No	No	Yes	Yes	No	No	No	No	SRT	5-10
6	-1.6		No	No	No	No	No	No	Yes	No	naive	N/A
7	-1.8		Yes	No	No	No	No	No	No	No	SRT	5-10
8	-2.2		Yes	No	Yes	Yes	Yes	No	No	No	SRT	1-5
9	-0.9		No	No	Yes	Yes #	No	No	No	No	SRT	5-10
10	-1.5		No	No	No	Yes	No	No	No	No	naive	N/A
						Osteoporosis						
1		-2.4	Yes	No	Yes	Yes	Yes	No	No	No	SRT	5-10
2	-3.2		No	No	Yes	Yes *	Yes	No	No	Yes	ERT	<10
3	-3.2		No	No	Yes	No	No	No	No	No	ERT	1-5
4		-2.7	No	No	Yes	Yes	Yes	Yes	No	No	SRT	<10
5	-2.8		No	No	No	Yes #	No	No	No	No	ERT	<10
6		-3.3	No	No	Yes	Yes #	Yes	No	No	No	naive	1-5
7	-2.7		Yes	Yes	Yes	Yes #	No	No	Yes	No	ERT/SRT	<10
8		-2.5	Yes	Yes	Yes	No	Yes	No	No	Yes	ERT	5-10
9	-3.5		No	Yes	Yes	No	Yes	No	No	No	ERT	5-10
10	-2.3		No	No	No	No	Yes	No	No	Yes	SRT	0-1
11	-2.3		No	Yes	Yes	No	No	No	Yes	No	ERT	<10
12	-2.2		No	Yes	Yes	Yes	Yes	No	Yes	Yes	ERT	1-5
13	-3.3		Yes	No	No	No	No	No	No	No	ERT	5-10
14	-4.5		No	Yes	No	Yes	No	No	No	No	SRT	5-10

Table S2.

	Total	Males	Females	Average Age
Controls	15	9	6	48±11
GD no bone complications	9	2	7	35±13
GD osteopenia	10	2	8	43±15
GD osteoporosis	14	4	10	47±14

Table S3.

ID	Age	Sex	Genotype
No bone complications (N)			
1	18	F	N370S/F213I
2	56	F	N370S/N370S
3	41	F	N370S/R463C
4	50	F	N370S/N370S
5	29	F	N370S/N370S
6	25	F	L444P/L444P
7	38	F	N370S/W381X
8	21	M	N370S/L444P
9	33	M	R48Q/L444P
Osteopenia (OSN)			
1	29	F	N370S/N370S
2	61	F	N370S/N370S
3	32	F	N370S/L444P
4	52	F	N370S/N370S
5	40	F	N370S/R463C
6	41	F	N370S/N370S
7	51	F	N370S/L444P
8	74	F	N370S/N370S
9	65	M	N370S/N370S
10	18	M	N370S/N370S
Osteoporosis (OSR)			
1	57	F	L444P/R502C
2	54	F	N370S/L444P
3	20	F	N370S/L444P
4	62	F	N370S/N370S
5	30	F	N370S/N370S
6	63	F	N370S/R496H
7	44	F	L444P/R493C
8	68	F	N370S/R463C
9	45	F	N370S/N370S
10	46	F	N370S/N370S
11	55	M	N370S/L444P
12	36	M	N370S/Y412X
13	39	M	N370S/N370S
14	42	M	N370S/N370S

Figure S1.

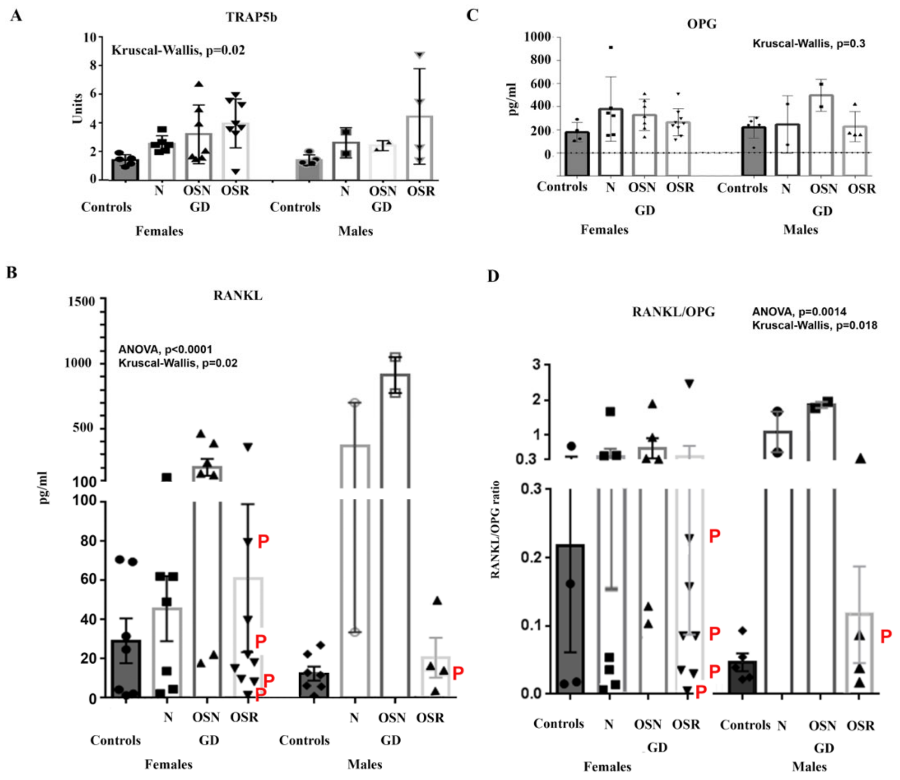

Supplemental Figure 1

Supplemental Figure S1. (**A)** TRAP5b concentrations in controls and GD females vs. males. GD groups were divided on N, OSN, and OSR. P<0.05 Kruskal-Wallis and Brown-Forsythe tests. Data are means ±SEM. Measurements (units/50 µl). (**B**) RANKL concentrations in controls and GD females vs. males. Red "P" marks patients treated with Prolia. GD groups were divided into N, OSN, and OSR. P<0.05 ANOVA and Kruskal-Wallis tests. Data are means ±SEM. Measurements of pg/ml. (**C)** OPG level in controls and GD females vs. males. GD groups were divided into N, OSN, and OSR. Data are means ±SEM. Measurements (pg/ml). **(D)** RANKL/OPG ratio in controls and GD females vs. males.P<0.05 ANOVA and Kruskal-Wallis tests.

Figure S2.

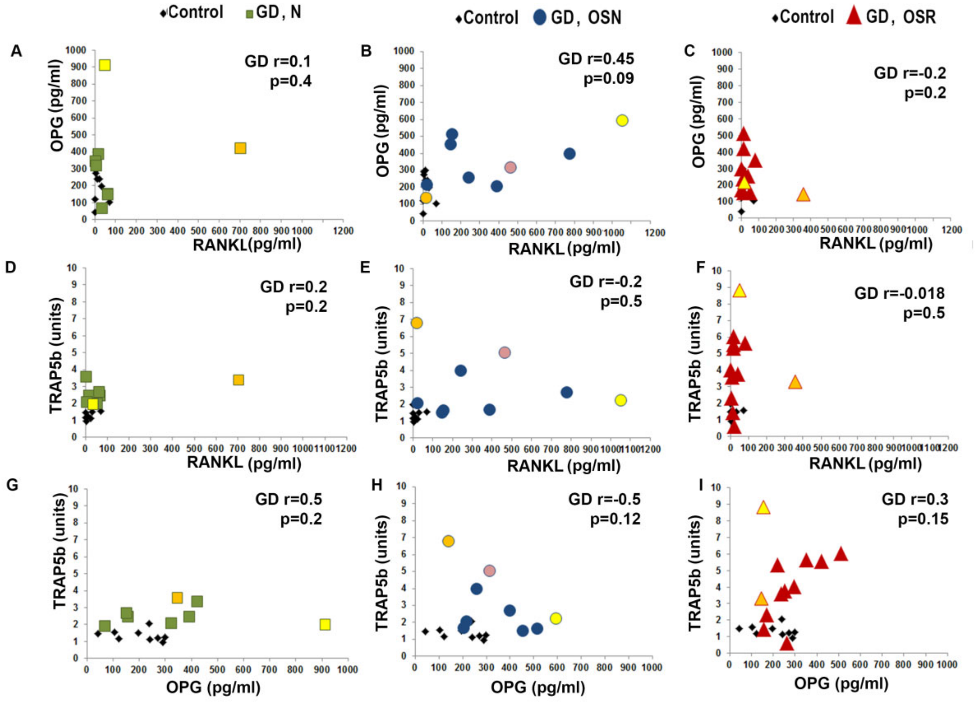

Supplemental Figure 2

Supplemental Figure S2. Scatterplots represent the correlation between RANKL, OPG, and TRAP5b. **(A)** Correlation between OPG and RANKL in the control group (black) and GD group without bone complication (N, green). The yellow and bright yellow squares represent the same GD patient in A, D, and G graphs. (**B**) Correlation between OPG and RANKL in the control group (black) and GD group with OSN (blue). The yellow, bright yellow and pink circles represent the same GD patients in B, E, and H graphs. (**C**) Correlation between OPG and RANKL in the control group (black) and GD group with OSR (red). Yellow, bright yellow triangles represent the same GD group in C, F, and I graphs. **(D)** Correlation between TRAP5b and RANKL in the control group (black) and GD group without bone complication (N, green). **(E)** Correlation between TRAP5b and RANKL in the control group (black) and GD patients with OSN. **(F)** Correlation between TRAP5b and RANKL in the control group (black) and GD patients with OSR. **(G)** Correlation between TRAP5b and OPG in the control group and GD group. **(H)** Correlation between TRAP5b and OPG in the control group and GD patients with OSN. **(I)** Correlation between TRAP5b and OPG in the control group and GD group with OSR. There was no linear correlation between TRAP5b and RANKL or OPG levels in GD (Pearson's linear regression correlation analysis, right top corner of the graphs: R and P values).

Figure S3.

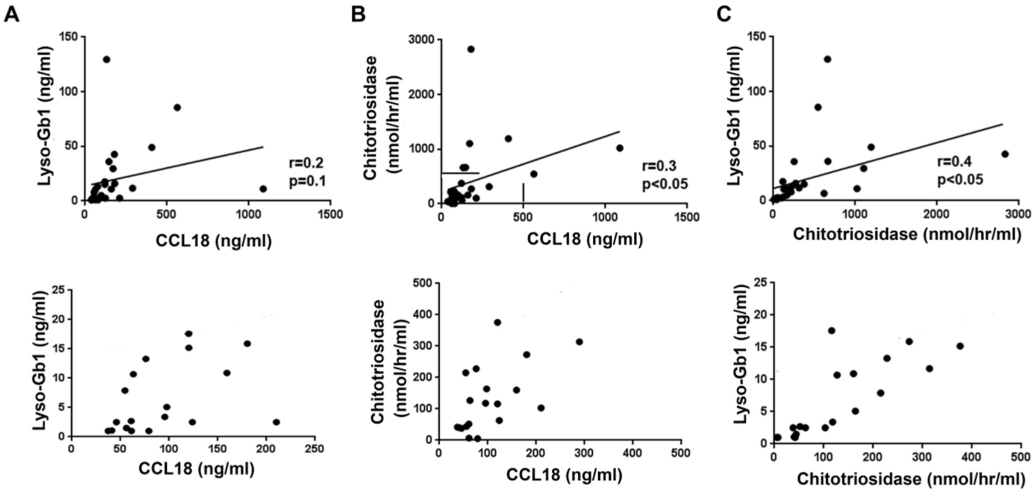

Supplemental Figure 3

Supplemental Figure S3. Scatterplots represent the correlation between Lyso-Gb1, CCL18, and chitotriosidase (CHITO) in all GD patients.

(A) Correlation between CCl18 and Lyso-Gb1 in GD. Original scatterplot (top graph) and with the lowest range of CCL18 0-250 ng/ml and Lyso-Gb1 0-25 ng/ml scatterplot (bottom). **(B)** Correlation between CHITO and CCL18 in GD patients. Original scatterplot (top graph) and with the lowest range of CHITO and CCL18: 0-500 ng/ml scatterplot (bottom). **(C)** Scatterplot between CHITO and Lyso-Gb1 in GD. Original scatterplot (top graph) and the same scatterplot with the lowest range of CHITO 0-500 nmol/hr/ml and Lyso-Gb1 0-25 ng/ml (bottom). P<0.05 statistically significant results. Statistical comparison was determined via Pearson's linear regression analysis, r= correlation coefficient.

Figure S4.

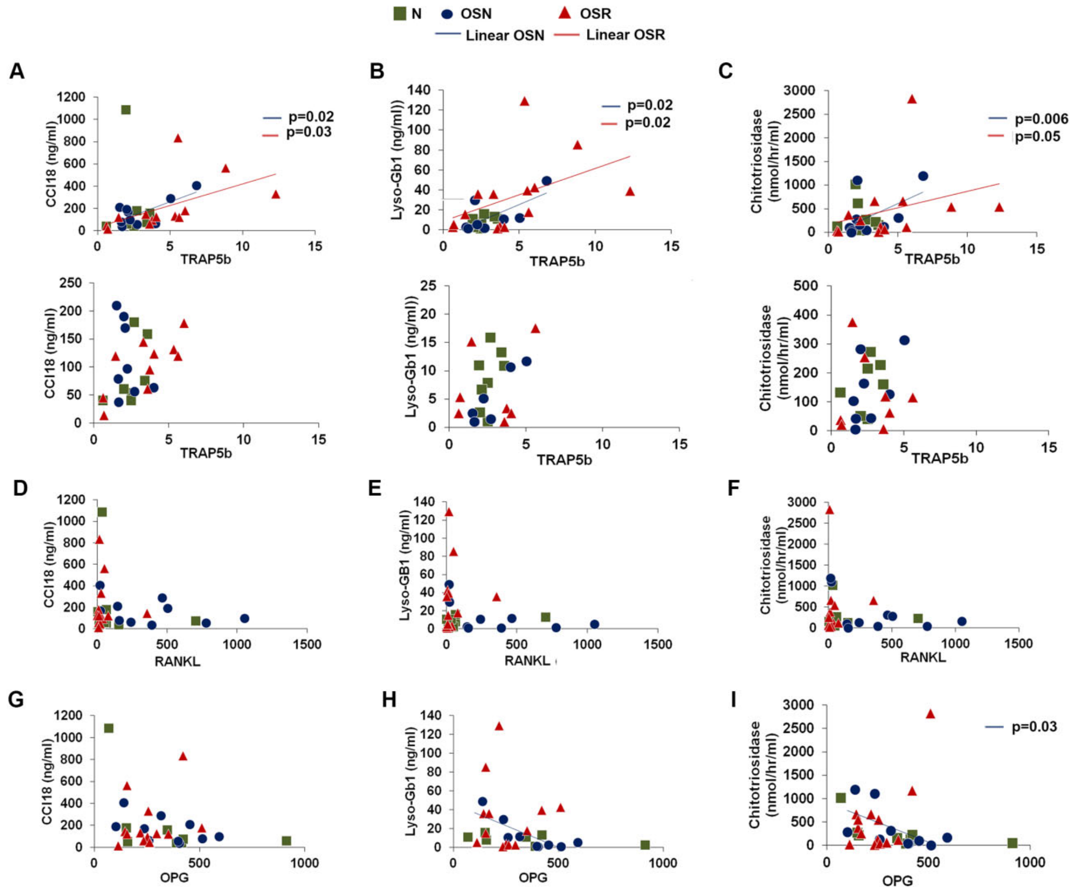

Supplemental Figure 4

Supplemental Figure S4. Scatterplots represent the correlation between Lyso-Gb1, CCL18, chitotriosidase (CHITO) and bone biomarkers.

(A) Correlation between CCl18 and TRAP5b in GD group without bone complication (N, green), OSN (blue) and OSR (red). Original scatterplot (top graph) and with lowest range of CCL18 0-250 ng/ml scatterplot (bottom). **(B)** Correlation between Lyso-Gb1 and TRAP5b in GD group without bone complication (N, green), OSN (blue) and OSR (red). Original scatterplot (top graph) and with the lowest range of Lyso-Gb1 0-25 ng/ml scatterplot (bottom). **(C)** Scatterplot between CHITO and TRAP5b in GD groups: (N, green), OSN (blue) and OSR (red). Original scatterplot (top graph) and the same scatterplot with the lowest range of CHITO 0-500 nmol/hr/ml (bottom). **(D-E-F)** Correlation between CCl18 (D), Lyso-GB1 (E), CHITO (F) and RANKL in GD groups: N (green), OSN (blue) and OSR (red). **(G-H-I)** Correlation between CCl18 (G), Lyso-GB1 (H), CHITO (I) and OPG in GD groups: N (green), OSN (blue) and OSR (red). $P<0.05$ statistical comparisons were determined via Pearson's one tail linear regression correlation analysis.
